# Effects of Phytoecdysteroids (PEDS) Extracted from *Cyanotis arachnoidea* on Rumen Fermentation, Enzyme Activity and Microbial Efficiency in a Continuous-Culture System

**DOI:** 10.1371/journal.pone.0153584

**Published:** 2016-04-15

**Authors:** Deyong Li, Yawei Zhang, Zhenliang Cui, Liwen He, Wanbao Chen, Qingxiang Meng, Liping Ren

**Affiliations:** State Key Laboratory of Animal Nutrition, College of Animal Science and Technology, China Agricultural University, Beijing, P. R. China; National Renewable Energy Lab, UNITED STATES

## Abstract

The objective of this study was to evaluate the effects of supplementation of phytoecdysteroids (PEDS) extracted from *Cyanotis arachnoidea* on rumen fermentation, enzymes activity and microbial efficiency in a dual flow continuous-culture system. A single-factor experimental design was used with twelve fermenters in 4 groups with 3 replicates each. Fermenters were incubated for a total of 7 days that included first 4 days for adaptation and last 3 days for sampling. PEDS was added at levels of zero (as control), 5, 10, and 15 mg/g of the substrate (DM). The results showed that increasing supplementation levels of PEDS resulted in incremental digestibility of dry matter (DMD) (quadratic, P = 0.001) and organic matter (OMD) (quadratic, P = 0.031), but unchanged digestibility of neutral detergent fiber (NDFD), crude protein (CPD) and acid detergent acid (ADFD). As supplementation levels of PEDS increased, there were decreased response in the concentration of ammonia nitrogen (NH_3_-N) (linear, P = 0.015) and increased response in molar proportions of butyrate (linear, P = 0.004), but unchanged response in total volatile fatty acid (TVFA) and the molar proportion of acetate and propionate, respectively. Increasing PEDS supplementation levels decreased the ratio of acetate to propionate (linear, P = 0.038), suggesting an alteration of rumen fermentation pattern occurring due to PEDS supplementation in the diet. Supplementation of PEDS significantly increased activities of glutamate dehydrogenase (quadratic, P = 0.001), alanine dehydrogenase (quadratic, P = 0.004), glutamate synthase (linear, P = 0.038), glutamine synthetase (quadratic, P = 0.011), respectively. There were no discernible differences in the activity of carboxymethyl cellulose (CMCase), xylanase and protease regardless of the treatments. The daily production of microbial nitrogen (linear, P = 0.002) and microbial efficiency (MOEEF) (linear, P = 0.001) increased linearly as supplementation levels of PEDS increased. The decreased response of fluid NH_3_-N and the increased response of MN indicated that PEDS positively increased the synthesis of microbial proteins.

## Introduction

Ruminants are able to digest almost all types of roughage due to their special digestive system, particularly through the process of rumen fermentation. The end products of rumen fermentation, which are primarily volatile fatty acids (VFA) and microbial crude protein (MCP), provide almost 80% of the host's energy requirements and 60–85% of the protein requirements [1]. The modulation of rumen fermentation is a priority in improving ruminant production efficiency. Antibiotics, such as monensin, tylosin and others, have been widely applied in ruminant production since the 1950s as a strategy to improve feed conversion rates and production performance while reducing costs [2, 3]. However, with these remarkable improvements, the abuse of antibiotics has resulted in plenty of problems relating to resistance and residues that have become increasingly serious [4, 5].

The use of natural plant compounds as substitutes for antibiotics has emerged due to the health threat from residual antibiotics and the occurrence of multi-drug-resistant bacteria in food, including animal products, and the practice has been reinforced by bans on the use of antibiotics. Hence, the search for natural alternative antibiotics has been regarded as highly important for the use of feed additives in animal farming as well as for human health. In particular, plant extracts have received much attention in the development of alternative antibiotics because of their characteristics of natural, no-residue and their biological activities [6, 7].

As a type of plant extract, ecdysteroids are steroids with 27, 28 or 29 carbon atoms and skeletons characterized by hydroxyl groups, and thus far, more than 150 ecdysteroids have been identified in plants [8]. The main zoo-ecdysteroid is 20-hydroxyecdysone (20E), which is recognized as the most biologically active ecdysteroid [9]. Phytoecdysteroids (PEDS), ecdysteroid analogs, occurring in approximately 5–6% of all plant species, especially ferns and chenopodiaceous and compositae plants, primarily ponasterones and makisterone C [10, 11]. In recent years, PEDS and their analogs have been widely explored for using as agricultural, biological, and pharmacological regulators due to their multiple active ingredients [12–14]. Additionally, there were a few reports about the positive effects of these compounds on muscle and liver protein synthesis [15, 16] and animal growth performance [17, 18]. Some researchers [19, 20] found that the extracts of *Asparagus dumosus* and *Serratula coronata* L. were resistant to several bacteria and fungi due to the presence of 20E.

However, the literature contains little information about the potential effects of PEDS on ruminants, such as through altering rumen fermentation and metabolism. A researcher reported that ecdysteroids decreased the concentration of ammonia nitrogen (NH_3_-N) and increased the concentration of total volatile fatty acid (TVFA) *in vitro* [21]. Based on our previous unpublished results of PEDS supplementation on *in vitro* fermentation, we hypothesized that PEDS showed a strongly positive effect on rumen fermentation and digestion, especially on nitrogen (N) metabolism. Therefore, the present study was conducted to test this hypothesis and determine the influence of PEDS on rumen fermentation, digestion and microbial efficiency over 7 days of incubation in a dual flow continuous-culture system.

## Materials and Methods

### Continuous culture system

A dual-flow continuous-culture system was used in this study. The basic apparatus was consisted of 12 independent 780 mL fermenter flasks which are immersed in a waterbath heated by a thermostatically controlled heater (Model CTL-7, Tinko Instrument Co., Ltd, Suzhou, China) and automatically stirred by a mini-motor (Model YYCJM, Ningbo Instrument Motor Factory, Ningbo, China). The fermenter was a polycarbonate jar which was equipped on the top cover with several input ports for buffer infusion, CO_2_ purging, pH monitoring and feed automated-feeding, and also with an effluent outlet at a height allowing a liquid volume of 560 mL within the fermenter before overflow. Constant-flow pumps (Model BT100-1L, Longerpump Co. Ltd, Baoding, China) were used for delivering buffer solution from buffer reservoirs into each of fermenters. An automated feeding device, mounted above each of the fermenter, was used to deliver pelleted diets into each fermenter.

### Diets and collection of rumen fluid

The animals used as rumen fluid donors in this study were maintained at the Research Base of the Beef Cattle Research Center, China Agricultural University, strictly according to the Regulations for Laboratory Animals of Beijing (Revised Edition, 2004). The protocol was approved by the Animal Welfare Committee of the China Agricultural University (Permit Number: DK1008). Four Limousin steers (approximately 550 kg average body weight) installed with permanent rumen cannula were used as donors of the rumen fluid. The steers were gradually adapted to the experimental diet (Table 1) formulated to meet the steers’ requirements (NRC, 2000) over a period of two weeks before the fluid was collected. The rumen contents were taken from four steers via ruminal fistulas before morning feedings, uniformly blended by a Milk-fat blender (1 min, Model DM-5L-B, Xuzhou Huijia Equipment Factory, China) and then strained through two layers of cheesecloth in an anaerobic environment. The liquid fraction was immediately inoculated into each of 12 pre-warmed (39°C) fermenters.

**Table 1:** The ingredients and chemical composition of the basal diet.

Ingredients	g/kg dry matter (DM)
Steam-flaked corn	341
Soybean curb residue	126
Brewer's grains	81
Maize silage	390
Chinese wild rye grass	50
Sodium bicarbonate	3
Salt	5
Premix^a^	5
Chemical composition^b^	
OM	939.1
CP	130.2
ADF	292.7
NDF	538.6
Ca	6.12
P	2.93

^a^ Premix contained (per kg): vitamin A, 3000 IU; vit. D_3,_ 1200 IU; vit. E, 10 IU; Cu, 8 mg; Fe, 50 mg; Zn, 30 mg; Mn, 40 mg; Co, 0.1 mg; Se, 0.2 mg; I, 0.5 mg.

^b^ OM = organic matter, CP = crude protein, ADF = acid detergent fiber, NDF = neutral detergent fiber.

### Continuous culture operations

PEDS was extracted from *Cyanotis arachnoidea* (Kingsci Biotechnology Co., Ltd., Shanxi, China). The main active ingredients are β-ecdysone and 20E with the content of 95% measured by high performance liquid chromatography.

A single-factorial experimental design was used, and zero (control), 5, 10 and 15 mg of PEDS per g substrate (based on DM) was added, respectively. Twelve fermenters were divided into 4 groups with 3 replicates each. On the first day of the period, all fermenters were inoculated with ruminal fluid obtained from the four steers. Artificial saliva containing 0.4 g/L urea to simulate recycled N was continuously infused into fermenters [22]. The dilution rates of solids and liquids of fermenters were 5%/h and 10%/h, respectively, by regulation of the buffer input and filtrate removal. Fermenters maintained at a constant temperature of 39°C and were continually purged with CO_2_ at a rate of 40 mL/min to preserve anaerobiosis.

Fermenters were operated for two 7-d periods consisting of a 4-d diet adaptation period followed by a 3-d sampling period. The substrate for each group was the same as the diet of the steer and mixed with PEDS according to the addition levels, then pelleted for convenient delivery. Each fermenter was supplied with 48 g DM of the diet in 4 equal portions over a 24-h period through an automated feeding system.

### Sample collection and processing

During the 3-day sampling period, the effluent fluid and fermenter contents were sampled once daily at 3 h after the first feeding. The fermenter contents used for the determination of VFA and NH_3_-N were mixed with 20% (v/v) sulfuric acid solution and then stored at -20°C until analysis. The samples used to determine the enzyme activity were taken and immediately stored in a -80°C freezer. Liquid and solid effluent was recorded daily and discarded until the last 3 days of each period, when it was collected, mixed with a 37% formaldehyde solution at a rate of 2.5% (v/v) and completely homogenized. During homogenization, 1 500-mL subsample of the effluent was collected; the subsamples were centrifuged at 20,000 g for 30 min at 4°C, washed twice with distilled water (20,000 g for 30 min at 4°C) and then lyophilized for chemical analysis and the calculation of true digestibility. On the last day of each period, the remaining contents of each fermenter were collected and mixed with a 37% formaldehyde solution at a rate of 2.5% (v/v) and vigorously blended for 1 min. After being strained through 4 layers of cheese cloth, the fluid fraction was centrifuged at 500 g for 5 min to remove the feed particles and the supernatant fluid was re-centrifuged at 20,000 g for 30 min at 4°C. The sediments were washed three times with 0.9% (w/v) saline solution (first time) and distilled water (second and third times) by centrifugation (20,000 g for 30 min at 4°C), and the resulting pellet was lyophilized for the measurement of purines.

### Chemical analysis

The moisture (934.01) and ash (942.05) contents of the diets were determined by oven drying at 95–100°C based on the AOAC (2012) [23], and the true digestibility of DM and OM was then calculated. Crude protein (CP) was determined by the Dumas method (968.06) using N Analyzer (Rapid N III, Elementar, Germany), and calculated as N × 6.25 [23]. Neutral detergent fiber (NDF) and acid detergent acid (ADF) of the feed and effluent were measured following the method described by Van Soest et al. [24] using an ANKOM^220^ Fiber Analyzer (ANKOM Technology Corp., A220, 230V~50/60Hz, 8A, Macedon, NY, USA).

The pH value of the fermenter content was measured 3 h after the first feeding using a pH meter (Testo 205, Testo A G, Germany), and NH_3_-N was analyzed by visible spectrophotometry (UV-VIS8500, Tianmei, Shanghai, China) following the procedure of Broderick and Kang [25]. VFA was determined by the gas chromatography (Varian Model 3400, Varian Instrument Group, Walnut Creek, CA, USA) [26]. The activities of xylanase and CMCase were measured according to the procedure of Miller et al. [27] and expressed as μmole of reducing sugars produced per min per mL under the conditions of the assay. For the measurement of protease activity, the reaction mixture contained 1 mL enzyme and 1 mL 10 g/L casein and was incubated at 40°C for 10 min. The reaction was stopped by adding 3 mL of 0.4 mol/L trichloroacetic acid, and the tyrosine was measured [28]. The activity of glutamate dehydrogenase (GDH), glutamine synthetase (GS), alanine dehydrogenase (ADH) and glutamate synthase (GOGAT) were measured using the reagent kits (Beijing Sino-UK Institute of Biological Technology, Beijing, China) and determined by the Auto-Biochemical Analyzer (7180, Hitachi Limited, Tokyo, Japan). RNA of the isolated microorganisms and effluent residues were determined according to the procedure of Zinn and Owens [29]. The daily production of non-microbial nitrogen (NMN) and microbial nitrogen (MN) then were calculated. Microbial efficiency (MOEEF) was expressed as g of MN per kg of OM truly digested (OMTD).

### Statistical analysis

The results were analyzed in accord with a completely randomized design and data were determined by analysis of variance using the ANOVA procedure in SAS (Version 9.0 Edition, 2002) to account for the addition of PEDS (i.e., 0 vs. 5g/kg vs. 10g/kg vs. 15g/kg) as the main effects, and fermenters, sampling times and incubation period as random effects. Linear and quadratic orthogonal contrasts were tested using the CONTRAST statement of SAS with coefficients estimated based on the PEDS supplementation. The differences among means with P<0.05 were considered significant and those between 0.05 and 0.10 were considered tendency.

## Results

### The effects of PEDS on the digestibility in continuous-culture

The digestibility of nutrients is presented in Table 2. The digestibility of DM and OM increased when 5 and 10 mg/g PEDS was included in the diet compared with the control diet and decreased when 15 mg/g PEDS was included in the diet. A quadratic effect was found for digestibility of DM and OM (P<0.05) and the highest values were observed at the level of 10 mg/g of PEDS. There were no discernible effects of PEDS supplementation on the true digestibility of NDF, ADF and CP, respectively (P>0.05), while when the PEDS supplementation at 10 mg/g, the digestibility of CP showed the maximum performance.

**Table 2:** The effects of PEDS on the digestibility of the fermentation substrate in continuous-culture (mg/g)

Items	PEDS supplementation level (mg/g)				SEM^b^	P-value	
	0	5	10	15		Linear	Quadratic
Digestibility (mg/g)^a^							
DMD	651.3	702.0	732.1	648.1	13.5	0.069	0.013
OMD	687.6	702.9	728.6	671.2	12.8	0.702	0.031
NDFD	463.6	456.4	455.6	436.8	8.5	0.648	0.133
ADFD	467.1	476.4	456.1	453.8	4.80	0.515	0.465
CPD	442.6	443.0	487.1	425.0	18.4	0.911	0.113

^a^ DMD = dry matter digestibility, OMD = organic matter digestibility, NDFD = neutral detergent fiber digestibility, ADFD = acid detergent fiber digestibility, CPD = crude protein digestibility

^b^ SEM: Standard error of means, where n = 3 per treatment.

### The effects of PEDS on the activity of enzymes

Increasing the supplementation of PEDS showed no significant effects on the activity of CMCase, xylanase and protease (P>0.05), but the maximum values of CMCase and xylanase activity were observed at the level of 10 mg/g PEDS, respectively. Increasing the supplementation of PEDS significantly increased the activity of GDH (quadratic, P<0.05) and ADH (quadratic, P<0.05) and the highest values were observed at 10 mg/g of PEDS supplementation level. The activity of GS increased linearly and quadratically (linear, P<0.05; quadratic, P<0.05) along with the PEDS supplementation level increased and the maximum value was obtained at the level of 10 mg/g. The supplementation of PEDS linearly increased the activity of GOGAT (linear, P<0.05), but the best performance was from 10 mg/g. Based on the results above, the supplementation of 10 mg/g was the optimum addition level.

### The effects of PEDS on rumen fermentation traits

Increasing the supplementation of PEDS showed no significant effects on fermenter pH values (P>0.05) and the TVFA concentration (P>0.05), though the maximum value of TVFA concentration was observed from 10 mg/g. As supplementation level of PEDS increased, there was significantly decreased response in the concentration of NH_3_-N (linear, P<0.05). For individual fatty acid, increasing PEDS supplementation had no significant effects on the proportion of acetate (P = 0.066) and propionate (P = 0.092), though there were obvious change tendency. There was a linearly increased response in the proportion of butyrate (linear, P<0.05), while the A: P ratio decreased linearly along with the supplementation of PEDS (linear, P<0.05).

### The effects of PEDS on nitrogen metabolism and MOEEF

**Fig 1:**
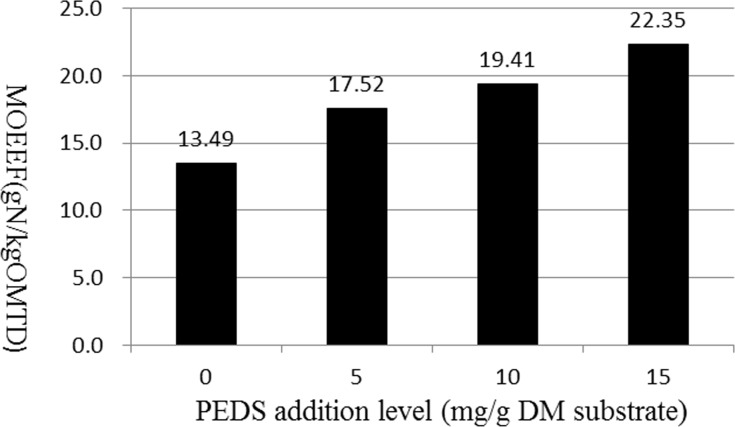
The microbial efficiency affected by the addition level of PEDS in continuous culture. (A) The addition level of PEDS was 0 (control), 5, 10 and 15 mg/g DM substrate. (B) MOEEF (microbial efficiency) was expressed as g of microbial nitrogen per kg of OM truly digested (OMTD).

The daily production of NH_3_-N decreased linearly (linear, P<0.05) following the increase of the addition levels of PEDS. As PEDS supplementation levels increased, there was no visible effect of PEDS on daily production of non-microbial nitrogen (NMN) (P>0.05), while the daily production of microbial nitrogen (MN) increased linearly (P<0.05). Fig 1 showed the effects of the addition level of PEDS on the microbial efficiency (MOEEF) and the variation of MOEEF presented a linear pattern in response to the increasing PEDS levels (linear, P<0.05).

## Discussion

### Digestibility of substrate

Antimicrobial properties of natural plant extracts may provide an alternative way to manipulate ruminal fermentation for improvement of energy and protein utilization in ruminants. Phytoecdysteroids are a type of plant extract that mainly exists in *Cyanotis arachnoides*, and the present study showed that PEDS had a positive effect on the digestibility of DM and OM (Table 2). However, literature about the effect of PEDS on rumen fermentation was rather absent except some researchers reported that pharmacological and biological functions of plants contain such a PEDS component [12, 20]. Wei et al. proved that ecdysteroids (an analog of PEDS) increased gas production *in vitro* when added to the substrate at a level of 0.2 mg/g [21]. Though there was no available data in their research about the digestibility of nutrients, Meken et al. demonstrated that gas production was positively related to the digestion of substrates [30].

Microbial digestion involves the integrated action of multiple enzymatic activities and Minato et al. reported that in rumen 91% of endoglucanase and 88% of xylanase were produced by the microbes adhered to the feed particles [31]. Therefore, digestion of nutrients, especially the NDF and ADF closely depends on increases of the population of cellulolytic bacteria and activities of cellulases, such as CMCase, xylanase and β-glucosidase. In the current study, PEDS showed no significant effects on the activity of CMCase and xylanase (Table 3) and the digestibility of NDF and ADF (Table 2), but the group of 10 mg/g obtained the maximum numerical results of these parameters and further research needs to be conducted to confirm the optimum additive dosage of PEDS. Moreover, although there were no available data regarding the effect of PEDS upon microbial flora, previous studies reported that ecdysteroids had antimicrobial properties [32] and many plants containing ecdysteroids also exhibited antimicrobial activity [19, 20]. Our current study is rather primary, but the results first time prove that PEDS has a positive effect on the digestibility of nutrients and rumen cellulase activity.

**Table 3:** The effects of PEDS supplementation level on the activity of enzymes in continuous culture.

Items^b^	PEDS supplementation level (mg/g)				SEM^c^	P-value	
	0	5	10	15		Linear	Quadratic
CMCase^a^	0.100	0.193	0.206	0.185	0.051	0.115	0.076
Xylanase	0.421	0.424	0.522	0.481	0.046	0.143	0.591
Protease	11.15	11.21	10.48	10.11	0.302	0.337	0.527
GDH^a^	1.566	1.988	2.337	1.582	0.110	0.441	0.001
GS^a^	0.365	0.481	0.514	0.453	0.030	0.042	0.011
ADH^a^	0.739	0.876	1.019	0.685	0.058	0.938	0.004
GOGAT^a^	0.421	0.461	0.750	0.711	0.112	0.038	0.698

^a^ CMCase = carboxymethyl cellulose, GDH = glutamate dehydrogenase, GS = glutamine synthetase, ADH = alanine dehydrogenase, GOGAT = glutamate synthase.

^b^ Units of enzyme activity are as follows: CMCase (μmol glucose min^-1^mL^-1^), xylanase (μmol xylose min^-1^mL^-1^), and protease (μg tyrosine min^-1^mL^-1^), GDH (μmol glutamate min^-1^L^-1^), GS (μmol glutamine min^-1^mL^-1^), ADH (μmol L^-1^), GOGAT (μmol glutamate min^-1^L^-1^).

^c^ SEM: Standard error of means, where n = 3 per treatment.

### Fermentation traits

VFA, as the end products of rumen microbial fermentation, represent the main supply of metabolizable energy (70–80%) for the ruminants. As a result, any reduction in ruminal VFA production would be nutritional unfavorable for the animal. However, the TVFA concentration in the present study was not affected by the PEDS supplementation, suggesting that supplementation of PEDS may not alter the dietary fermentation and energy availability in the rumen. This result was totally consistent with other observation that no obvious effect of ecdysterone supplementation on ruminal TVFA concentration was found [21]. The production of VFA in the rumen is affected by multiple factors that mainly include the dietary digestion, feed intake and rumen microflora [33]. In the present study, the dose of PEDS supplementation at 10 mg/g had the maximum TVFA (85.52 mmol/L) that may result from the increased digestibility of OM and DM.

PEDS presented no significant effects on the proportion of acetate and propionate. However, the two parameters showed a tendency (0.5<P<0.1), resulted in a significant decrease in the ratio of acetate to propionate along with the supplementation level increase (Table 4). The production of acetate and propionate mainly depended on the in rumen microbial fermentation and the procedure was affected by plenty factors, among which the microbial flora played the dominant role. Therefore, rumen management was the most efficient method for ruminants and lots of regulators were applied in ruminant nutrient. Methane inhibitors, such as monensin could cause decreased acetate production and increased butyrate proportion in the rumen [34]. Our results (Table 4) are consistent with these conclusions, suggesting that PEDS, to some extent, may inhibit the methane production from rumen fermentation. A similar result was reported that the methane production was reduced by 2.9%, 14.9%, 16.5% when ecdysteroids was added *in vitro* and fermented for 12h, 24h and 48h, respectively [21]. Elimination of rumen protozoal growth by addition of PEDS may be responsible for such inhibition of methanogenesis in the *in vitro* rumen fermentation. Ruminal protozoa are known to provide hydrogen as a substrate for methanogens, and methanogenic archaea are metabolically correlated with ciliate protozoa [35, 36]. It has been reported that plenty of plant extracts affect methane production indirectly by suppressing the amount of protozoa [37]. Similarly, the ecdysone was found to improve the productivity of ruminants by elimination of rumen protozoal growth [12]. Furthermore, the linear increase models of molar proportion of butyrate and acetate in current study confirmed the hypothesis.

**Table 4:** The effects of PEDS on pH and rumen fermentation traits in continuous-culture.

Items	PEDS supplementation level (mg/g)				SEM^a^	P-value	
	0	5	10	15		Linear	Quadratic
pH	6.28	6.19	6.20	6.35	0.87	0.235	0.183
TVFA (mmol/L)	81.80	82.81	85.52	82.48	1.45	0.789	0.149
NH_3_-N (mg/dL)	18.53	17.63	18.08	16.81	0.45	0.015	0.320
Molar Proportion (%)							
Acetate	61.89	61.98	61.70	60.23	0.01	0.066	0.193
Propionate	25.72	26.72	26.78	27.83	0.09	0.092	0.343
Butyrate	8.67	8.49	9.36	9.34	0.10	0.004	0.677
A:P ratio^b^	2.39	2.39	2.32	2.15	0.05	0.038	0.110

^a^ SEM: Standard error of means, where n = 3 per treatment

^b^ A: P ratio = the ratio of molar proportion of acetate to propionate.

### Microbial efficiency

NH_3_-N is the major end product of dietary protein and non-protein nitrogen (urea and amino acids) as well as the main nitrogen source for microbial protein (MCP) synthesis by ruminal bacteria [38]. It is well recognized that NH_3_-N concentration alone cannot be used to assess microbial yield since both CP degradation and MCP synthesis occur simultaneously. Orskov found that the maximum microbe count was increased when the level of ruminal NH_3_-N ranged from 10 to 25 mg/dL [39], which consists with the present results (Table 5). It is generally accepted that the increase of protease activity was disadvantageous for ruminant nutrition for it may promote the degradation of good quality CP and produce more NH_3_-N, thus cause the waste of diet CP [40], while in the current study the PEDS did not alter the protease activity and the digestibility of CP. However, the observation that PEDS decreased the concentration of NH_3_-N in the current study suggested an increase in the efficiency of MCP synthesis reflected by the increased MN and MOEEF with the addition of PEDS (Table 5).

**Table 5:** The effects of PEDS on nitrogen metabolism and MOEEF in continuous culture.

Items	PEDS supplementation level (mg/g)				SEM^b^	P-value	
	0	5	10	15		Linear	Quadratic
N input (g/d)							
Diet N	1.06	1.05	1.05	1.06			
Urea N	0.47	0.47	0.47	0.47			
N output							
NH_3_-N (g/d)^a^	0.56	0.53	0.55	0.51	0.001	0.003	0.321
NMN (g/d)^a^	0.30	0.30	0.27	0.32	0.06	0.235	0.103
MN (g/d)^a^	0.52	0.59	0.64	0.76	0.06	0.002	0.164

^a^ NH_3_-N = ammonia nitrogen, NMN = non-microbial protein, MN = microbial nitrogen

^b^ SEM: Standard error of means, where n = 3 per treatment.

MCP contributes significantly to the CP requirement for ruminants and supplies an average of 59% (range from 34–89%) of non-ammonia nitrogen that passes to the duodenum [41]. Plenty of studies about natural plant extract enhancing the synthesis efficiency and production of MCP were reported in recent years [42, 43], while the literature, ether *in vitro* or *in vivo*, about PEDS used in this field was absent. It is well known that there are two main pathways illustrating the process of ammonia assimilation, which involved several key enzymes, such as GDH (the GDH pathway), GS and GOGAT (the GS-GOGAT pathway) [44, 45]. Other enzymes, such as ADH and asparagine synthetase (AS), have also been identified in ammonia assimilation by rumen bacteria [46]. Moreover, Chaudhary et al. confirmed that ecdysone significantly increased the activity of GDH in the rat brain [47].Therefore, based on the present results (Table 5), the mechanism through which PEDS increases the ruminal microbial efficiency in the current study may be the improvement of the activity of certain enzymes (GDH, GS and ADH).

## Conclusions

Supplementation of PEDS increased the digestibility of DM and OM, but significantly decreased the concentration of NH_3_-N. Activities of GDH, ADH, GOGAT and GS increased along with the supplementation of PEDS, though there were no discernible differences in the activity of CMCase, xylanase and protease. The daily production of MN and the value of MOEEF increased linearly as supplementation levels of PEDS increased. Based on the current results, when added at the level of 10 mg/g, PEDS enhanced the digestibility of OM for 5.96% and increased the microbial nitrogen for 23.08%, thus effectively improved the utilization efficiency of diet nitrogen and nutrients, which was beneficial and cost-effective for ruminants. Though the changes of the parameters did not show a consistent trend, PEDS showed a great potential for use as an effective feed additive.

## Supporting Information

S1 File The original data of the current research. The file includes 4 parts of original data of the current research, namely “The digestibility, The enzyme activity, Fermentation parameters, The nitrogen metabolism”.
(XLSX)
